# Plackett–Burman screening of physico-chemical variables affecting *Citrus* peel-mediated synthesis of silver nanoparticles and their antimicrobial activity

**DOI:** 10.1038/s41598-024-58102-x

**Published:** 2024-04-06

**Authors:** Bardees Mickky, Heba Elsaka, Muhammad Abbas, Ahmed Gebreil, Reham Shams Eldeen

**Affiliations:** 1 Botany Department, Faculty of Science, Mansoura University, Mansoura, 35516 Egypt; 2 Botany and Microbiology Department, Faculty of Science, Arish University, Arish, 45511 Egypt

**Keywords:** Antimicrobial, Orange peel, Plackett–Burman design, Silver nanoparticles, Antimicrobials, Nanobiotechnology

## Abstract

With the growing resistance of pathogenic microbes to traditional drugs, biogenic silver nanoparticles (SNPs) have recently drawn attention as potent antimicrobial agents. In the present study, SNPs synthesized with the aid of orange (*Citrus sinensis*) peel were engineered by screening variables affecting their properties via Plackett–Burman design. Among the variables screened (temperature, pH, shaking speed, incubation time, peel extract concentration, AgNO_3_ concentration and extract/AgNO_3_ volume ratio), pH was the only variable with significant effect on SNPs synthesis. Therefore, SNPs properties could be enhanced to possess highly regular shape with zeta size of 11.44 nm and zeta potential of − 23.7 mV. SNPs purified, capped and stabilized by cloud point extraction technique were then checked for their antimicrobial activity against *Bacillus cereus*, *Listeria innocua*,* Listeria monocytogenes*, *Staphylococcus aureus*, *Enterobacter cloacae*,* Escherichia coli*, *Klebsiella pneumoniae*, *Pseudomonas aeruginosa*, *Salmonella typhimurium* and* Candida albicans*. The maximum antimicrobial activity of SNPs was recorded against *E*.* coli*, *L*.* monocytogenes* and *C*. *albicans* with clear zone diameter of 33.2, 31.8 and 31.7 mm, respectively. Based on minimum inhibition concentration and minimum bactericidal concentration of SNPs (300 mg/l) as well as their effect on respiratory chain dehydrogenases, cellular sugar leakage, protein leakage and lipid peroxidation of microbial cells, *E*.* coli* was the most affected. Scanning electron microscopy, protein banding and DNA fragmentation proved obvious ultrastructural and molecular alterations of *E*.* coli* treated with SNPs. Thus, biogenic SNPs with enhanced properties can be synthesized with the aid of *Citrus* peel; and such engineered nanoparticles can be used as potent antimicrobial drug against *E*.* coli*.

## Introduction

Orange (*Citrus sinensis* L.) is an important fruit crop used for both juicy fruit bulb and aromatic peel^1^. *Citrus* peel is the main by-product of its fruits; with rich profile of phytochemicals such as cellulose, hemicellulose, lignin, coumarins, phenols and others^2^. The presence of these active constituents allows orange peel extract to serve as perfect reducing, stabilizing and capping agent for the synthesis of various nanoparticles. In this regard, aqueous extract of *Citrus* peel was used to assist green synthesis of zinc oxide nanoparticles^3^, gold nanoparticles^4^ as well as silver nanoparticles (SNPs)^5^. More interestingly, the use of these nanomaterials in various biomedical applications has gained great attention owing to their unique advantageous properties^5^. Using for drug delivery and bio-imaging as well as being antimicrobial, anti-diabetic and anti-inflammatory are some of these applications^4^.

Recently, green synthesis of nanoparticles using plant wastes has attracted attention because it is eco-friendly, economic and simple^6^. However, there are some challenges that hinder the perfect utilization of green synthesis for obtaining nanoparticles. Among these, factors affecting biogenic synthesis of nanoparticles are crucial and can affect shape, size, yield and stability of the formed nanoparticles. Temperature, pH, concentration of the plant extract, concentration of metal precursor and incubation period are few factors to be named^7^. In this context, Plackett–Burman design (PBD) could provide the perfect solution to overcome this obstacle as it provides an effective method to identify the relevant variables to be used for optimization. PBD is one of the fractional factorial designs that can monitor large number of parameters with minimum number of experiments. PBD is thus a screening tool that belongs to design of experiment procedure; and it is in particular an economical approach that allows feasible screening of many variables to detect their main effects on certain response(s). Therefore, PBD could be used in optimizing the process parameters for green synthesis of SNPs with performing the minimum number of experimental runs; thus saving great deal of time, effort and cost^8^. Optimization is thus a must to obtain nanoparticles with the desired characteristics and performance. Earlier studies showed that optimization of SNPs’ synthesis could lead to higher yield and stability as well as smaller size with uniform shape when compared with non-optimized SNPs^9^.

The growing resistance of many microbes, and bacteria in particular, to most of the known antibiotics has drawn attention to the importance of testing nanomaterials as alternative to traditional antibiotics. SNPs showed marked antimicrobial activity; and they were even considered more effective than other metal nanomaterials in destroying microbes^10^. However, some limitations were recorded while using SNPs as antimicrobial agents; the most common of which is their possible cytotoxicity on human^11^. Although their antimicrobial efficacy has been previously studied, their precise mode of action is still unrevealed. Based on former observations, SNPs could harm bacterial cells by inducing cell shape alteration, increasing cellular membrane permeability and disturbing respiration^12^. Other studies linked antimicrobial activity of SNPs with oxidative stress resulted from overproduction of active oxygen species that target essential macromolecules within the cell^13^. Nevertheless, in depth research on the antimicrobial activity of biogenic SNPs is to somewhat limited; especially for engineered SNPs. Thus, the current study aimed at using *Citrus* peel extract to mediate the synthesis of SNPs. Physico-chemical variables affecting green synthesis of SNPs would be screened in a trial to optimize the properties of the as-prepared SNPs. More importantly, the current study aimed at assessing antimicrobial activity of the biogenic SNPs and revealing the mechanism by which they could affect microbes.

## Materials and methods

### Crude synthesis of SNPs

Aqueous extract was prepared from orange (*Citrus sinensis* L.) peel. In a boiling water bath, 5 g air dry peel ground to fine powder was incubated in distilled water for 30 min. The homogenate was centrifuged then filtered through Whatman No. 40 filter paper and the filtrate was raised to 100 ml. Reducing power and antioxidant activity of the extract were determined as described by Dorman and Hiltunen^14^ and Prieto et al.^15^, respectively. Ascorbic acid content of the extract was determined following Ogunlesi et al.^16^. The amount of reducing sugars and that of total phenols were determined as described by Sadasivam and Manickam^17^, while flavonoids content was determined following Dewanto et al.^18^.

For biogenic synthesis of SNPs, 20 ml extract was mixed with 80 ml AgNO_3_ (1 mM) in plugged flaks. The as-prepared reaction mixture had pH value of 5.6. The mixture was incubated for 24 h at 32 °C in dark with continuous shaking at 120 rpm. Formation of SNPs was indicated by color change and spectral analysis at 300–900 nm by UV–visible spectrophotometer (6705 Jenway, England). The obtained SNPs were further examined by transmission electron microscopy (JEM-2100 TEM, JEOL, Japan).

### Screening of variables affecting SNPs synthesis

PBD was followed to screen the effect of temperature, pH, shaking speed, incubation time, peel extract concentration, AgNO_3_ concentration and extract/AgNO_3_ volume ratio on SNPs synthesis. Design of experiment (DOE) was performed using Minitab® (version 18.1) software. For a design with 8 variables (7 physico-chemical variables plus a dummy factor), a matrix of 12 runs (plus a center point run) was employed. Variables were screened each at 3 levels; center point, high level and low level. Center point is the level used for crude synthesis, while high level and low level were calculated as center point plus and minus its half value, respectively (Table 1). Therefore, design matrix for 13 runs could be obtained (Table 2).

**Table 1:** Variables screened using Plackett–Burman design to optimize *Citrus* peel-mediated synthesis of SNPs.

Variable	Code	Unit	High level (1)	Centre point (0)	Low level (− 1)
Temperature	X1	°C	48	32	16
pH	X2	–	8.4	5.6	2.8
Shaking speed	X3	rpm	180	120	60
Incubation time	X4	hour	36	24	12
Peel extract concentration	X5	%	7.5	5	2.5
AgNO_3_ concentration	X6	mM	1.5	1	0.5
Extract/AgNO_3_ volume ratio	X7	–	3/8	2/ 8	1/8
Dummy factor	X8	–	–	–	–

**Table 2:** Plackett–Burman design matrix to screen variables affecting *Citrus* peel-mediated synthesis of SNPs.

Run	X1	X2	X3	X4	X5	X6	X7	X8
1	− 1	1	1	− 1	1	− 1	− 1	− 1
2	− 1	1	− 1	− 1	− 1	1	1	1
3	− 1	− 1	− 1	− 1	− 1	− 1	− 1	− 1
4	1	− 1	1	1	− 1	1	− 1	− 1
5	1	− 1	1	− 1	− 1	− 1	1	1
6	1	− 1	− 1	− 1	1	1	1	− 1
7	− 1	− 1	1	1	1	− 1	1	1
8	0	0	0	0	0	0	0	0
9	1	1	− 1	1	− 1	− 1	− 1	1
10	1	1	− 1	1	1	− 1	1	− 1
11	− 1	− 1	− 1	1	1	1	− 1	1
12	− 1	1	1	1	− 1	1	1	− 1
13	1	1	1	− 1	1	1	− 1	1

SNPs obtained from each run were analyzed for 4 responses; maximum absorption at wavelength of 400–500 nm (A_max_ at λ_400–500_), zeta size, zeta potential and concentration. A_max_ at λ_400–500_ was determined with spectroscopy, size and potential by zetametry (Nano-zs90 zeta analyzer, Malvern, UK), while concentration was determined by atomic absorption spectrometry. Based on the results obtained for the responses, factorial PBD was analyzed in terms of analysis of variance (ANOVA), coded coefficients and model summary. Thus, estimated effects and contribution % of variables, as well as normal probability plot and Pareto chart of the standardized effects of variables, could be graphically represented. Also, a first-order polynomial regression equation was concluded:

$$ {\text{Y}} = \upbeta 0 + \sum \upbeta {\text{i}} + {\text{Xi}}\;\left( {{\text{i}} = {1},{2},{3}, \ldots {\text{K}}} \right) $$

where Y is the response, β0 is the model intercept, βi is the regression coefficient, Xi is the variable and K is the number of variables. Furthermore, regression equation was shortened by considering only the significant variable(s).

### Purification and characterization of the optimized SNPs

To purify optimized SNPs, the reaction mixture was centrifuged at 11,270 *g* for 30 min. Half ml triton *x*-100 (5%) was then added to 9.5 ml supernatant, mixed well and incubated in water bath at 70 °C for 2 h. The mixture was centrifuged at 1960 *g* for 30 min, placed in ice bath then the aqueous phase was decanted. The obtained SNPs were characterized by high resolution TEM (an imaging mode of TEM that allows direct imaging of the atomic structure to reveal lattice spacing) and by zetametry (size and potential analysis). Moreover, the number of atoms per nanoparticle was calculated according to Kalishwaralal et al.^19^.

### Surveying antimicrobial activity of SNPs

Antimicrobial activity of SNPs was evaluated by disk diffusion method against *Bacillus cereus* (11778™), *Listeria innocua* (33090™), *Listeria monocytogenes* (19,115™), *Staphylococcus aureus* (6538™), *Enterobacter cloacae* (13047™),* Escherichia coli* (10536™), *Klebsiella pneumoniae* (10031™), *Pseudomonas aeruginosa* (9027™), *Salmonella typhimurium* (14028™) and *Candida albicans* (EMCC number-105). The concerned strains were mostly of animal origin and were obtained from Microbiological Resources Centre (MIRCEN), Faculty of Agriculture, Ain Shams University, Egypt. The tested microbes were adjusted to 1 × 10^6^ colony-forming units /ml and spread evenly on Luria–Bertani (LB) agar medium. Sterile paper disks saturated with SNPs (300 mg/l), gentamicin (positive control) and sterilized water (negative control) were used. After incubation at 37 °C for 24 h, diameters of clear zones were measured and statistically analyzed using CoHort/CoStat® (version 6.311) software.

### Effect of SNPs on biochemical features of the microbial cells

For the microbes most affected by SNPs, minimum inhibitory concentration (MIC) and minimum bactericidal concentration (MBC) were determined^20^. MIC was determined following the standard spectrophotometric dilution method on LB broth medium, while MBC was determined following the standard agar method using LB agar medium. In addition, tolerance level of the tested microbes towards SNPs was determined as the ratio of MBC to MIC^21^.

The effect of SNPs at MIC on respiratory chain dehydrogenases activity of the tested microbes was determined by iodonitrotetrazolium chloride method^22^. Also, the effect of SNPs on cellular sugar and protein leakage was determined by dinitrosalicylic acid and coomassie brilliant blue methods, respectively^17^. In addition, lipid peroxidation of the microbes was determined by malondialdehyde-thiobarbituric acid method^23^. Theses parameters were determined for SNPs-treated and untreated microbes; and the % change in each parameter was calculated and graphically represented.

### Effect of SNPs on ultrastructural and molecular features of the microbial cells

For the microbe most affected by SNPs, sample was grown on nutrient agar medium with SNPs applied using disk diffusion at MIC. Untreated group was prepared using sterilized water instead of SNP. Ultrastructure of cell surface was studied by scanning electron microscopy (JSM 6510-LV SEM, JEOL, Japan). Also, protein banding of the microbe was conducted via sodium dodecyl sulphate polyacrylamide gel electrophoresis (SDS-PAGE) using TriFast™ kit (Peqlab, VWR Co., UK). For DNA fragmentation, GeneJet™ genomic DNA kit (Thermo Fisher Scientific Co., USA). For protein and DNA, gel was imaged using gel documentation system (Geldoc-it, UVP, UK) and data were analyzed using Totallab® (version 1.0.1) software. Both gels were presented to clearly indicate major differences between lanes.

## Results and discussion

### Crude synthesis of SNPs

**Figure 1:**
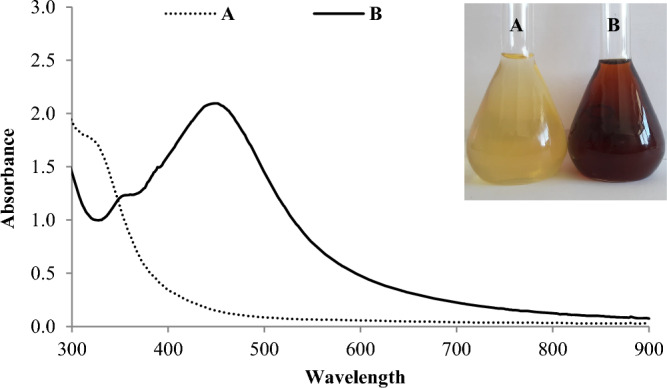
Visual observation and UV–visible spectroscopy of orange peel extract (**A**) and the resulted SNPs (**B**).

**Figure 2:**
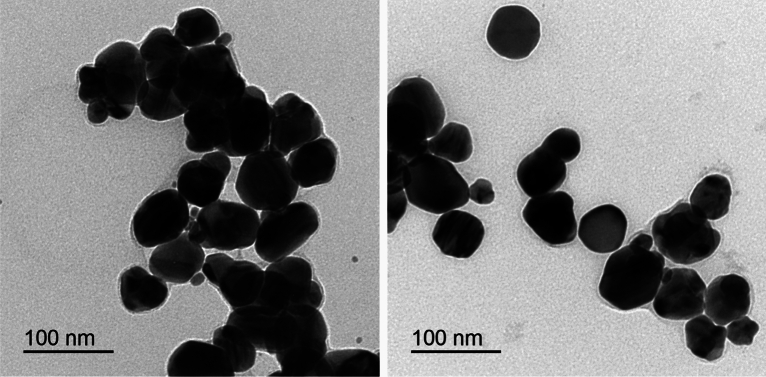
TEM of SNPs synthesized by the aid of *Citrus* peel extract.

Synthesis of SNPs from AgNO_3_ with the aid of *Citrus* peel was indicated by color change and spectral analysis of the reaction mixture. The mixture was changed from yellow to dark reddish brown; and it showed absorption peak at 450 nm (Fig. 1). Color change and absorption behavior can be ascribed to surface plasmon resonance of SNPs^24^. The ability of *Citrus* peel extract to mediate synthesis of SNPs can be attributed to the peel phytochemicals that promote reduction of silver ions into atoms. Silver atoms then self-assembled forming nuclei that ultimately grow into nanoparticle. Moreover, antioxidant activity of these phytochemicals can prevent the re-oxidation of silver atoms into ions; and thereby contribute to SNPs stability. Similar explanation of green synthesis of SNPs was previously postulated by Adrianto et al.^25^. Phytochemical analysis of *Citrus* peel extract revealed that it had reasonable amounts of ascorbic acid, reducing sugars, phenols and flavonoids (Table 3). These phytochemical are documented to possess marked reducing and/or antioxidant capacity^26^. This capacity is proven herein by the high value of reducing power and antioxidant activity of *Citrus* peel extract (Table 3). Characterization of the as-prepared SNPs by TEM revealed almost irregular shape with wide size ranging from 30 to 90 nm (Fig. 1). Shape, size and other features of SNPs are thought to be affected by the physico-chemical variables of crude synthesis (Fig. 2).

**Table 3:** Phytochemical content, reducing power and antioxidant activity and of *Citrus* peel aqueous extract (values listed are the mean of three replica ± standard deviation).

Ascorbic acid content (mg g^−1^ d wt)	1.00 ± 0.11
Reducing sugars content (mg g^−1^ d wt)	0.19 ± 0.10
Total phenols content (mg g^−1^ d wt)	14.37 ± 1.15
Flavonoids content (mg g^−1^ d wt)	2.26 ± 0.09
Reducing power (mg ascorbic acid equivalent ml^−1^)	0.79 ± 0.03
Antioxidant activity (mg ascorbic acid equivalent ml^−1^)	1.50 ± 0.01

### Screening of variables affecting SNPs synthesis

The effect of temperature, pH, shaking speed, incubation time, peel extract concentration, AgNO_3_ concentration and extract/AgNO_3_ volume ratio on A_max_ at λ_400–500_, size, zeta potential and concentration of SNPs was determined. Among the considered responses, the maximum value of A_max_ at λ_400–500_ was recorded for run 13 (Table 4). Run 13 was carried out by reacting 11.1 ml peel extract (7.5%) with 88.9 ml AgNO_3_ (1.5 mM) at 48 °C, pH 8.4 and 180 rpm for 12 h (Table 2). In addition, the minimum size of SNPs was recorded for run 2 (Table 4). Run 2 was carried out by reacting 27.3 ml peel extract (2.5%) with 72.7 ml AgNO_3_ (1.5 mM) at 16 °C, pH 8.4 and 60 rpm for 12 h (Table 2). Also, the minimum zeta potential was recorded for run 12 (Table 4). Run 12 was carried out by reacting 27.3 ml peel extract (2.5%) with 72.7 ml AgNO_3_ (1.5 mM) at 16 °C, pH 8.4 and 180 rpm for 36 h (Table 2). Furthermore, the maximum concentration was recorded for run 13 that caused the maximum A_max_ at λ_400–500_ (Table 4).

**Table 4:** Responses to variables affecting *Citrus* peel-mediated synthesis of SNPs.

Run	A_max_ at λ_400–500_	Zeta size (nm)	Zeta potential (mV)	Concentration (ppm)
1	1.653	33.11	− 18.50	33.55
2	1.575	22.02	− 20.70	80.33
3	0.584	609.40	− 10.20	28.46
4	0.058	598.40	− 4.66	11.04
5	0.709	831.20	− 3.26	22.10
6	1.493	734.80	− 4.16	84.53
7	1.001	678.80	− 2.32	26.25
8	2.215	84.87	− 18.20	69.56
9	1.437	42.22	− 23.00	29.78
10	2.828	42.15	− 17.60	21.67
11	0.624	555.80	− 7.33	48.77
12	2.304	45.47	− 23.70	75.70
13	3.535	32.20	− 22.80	116.26

**Figure 3:**
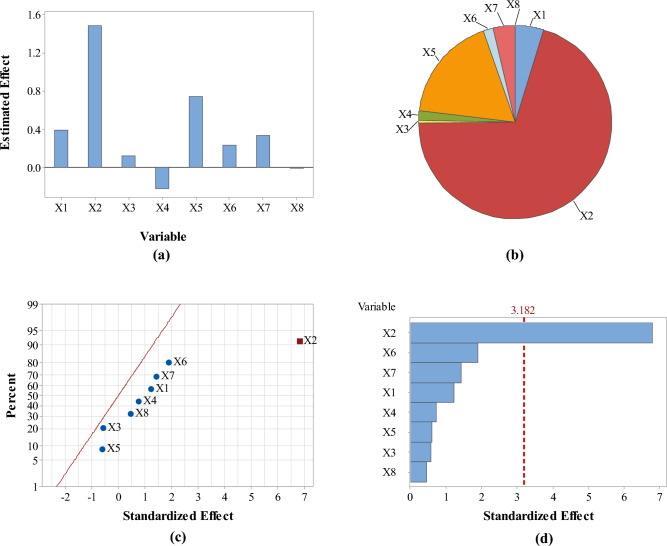
Estimated effects (**a**), contribution % (**b**), normal probability plot of the standardized effects (**c**) and Pareto chart of the standardized effects (**d**) of variables affecting *Citrus* peel-mediated synthesis of SNPs (the first response: A_max_ at λ_400–500_). Variables studied are temperature (X1), pH (X2), shaking speed (X3), incubation time (X4), peel extract concentration (X5), AgNO_3_ concentration (X6), extract/AgNO_3_ volume ratio (X7) and dummy factor (X8).

**Figure 4:**
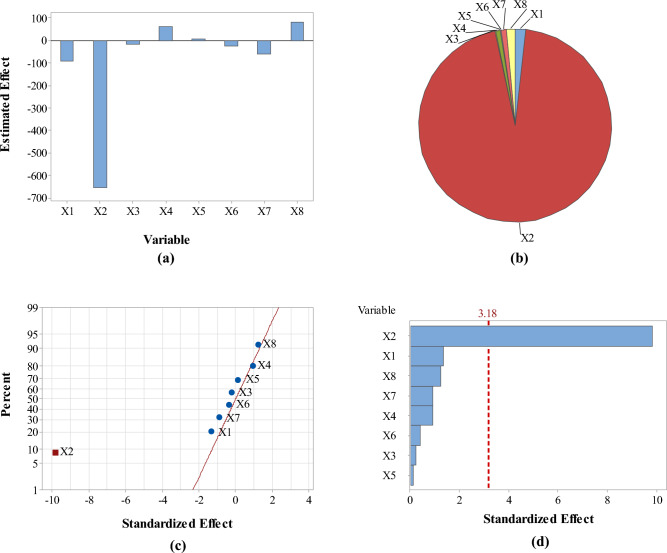
Estimated effects (**a**), contribution % (**b**), normal probability plot of the standardized effects (**c**) and Pareto chart of the standardized effects (**d**) of variables affecting *Citrus* peel-mediated synthesis of SNPs (the second response: zeta size in nm). Variables studied are temperature (X1), pH (X2), shaking speed (X3), incubation time (X4), peel extract concentration (X5), AgNO_3_ concentration (X6), extract/AgNO_3_ volume ratio (X7) and dummy factor (X8).

**Figure 5:**
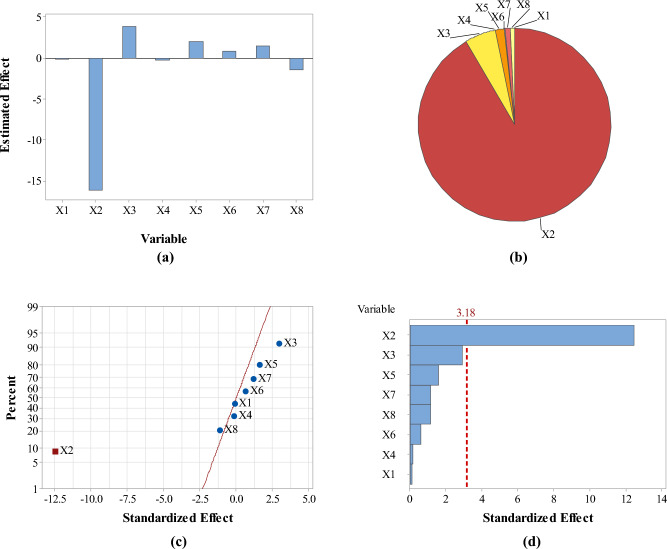
Estimated effects (**a**), contribution % (**b**), normal probability plot of the standardized effects (**c**) and Pareto chart of the standardized effects (**d**) of variables affecting *Citrus* peel-mediated synthesis of SNPs (the third response: zeta potential in mV). Variables studied are temperature (X1), pH (X2), shaking speed (X3), incubation time (X4), peel extract concentration (X5), AgNO_3_ concentration (X6), extract/AgNO_3_ volume ratio (X7) and dummy factor (X8).

**Figure 6:**
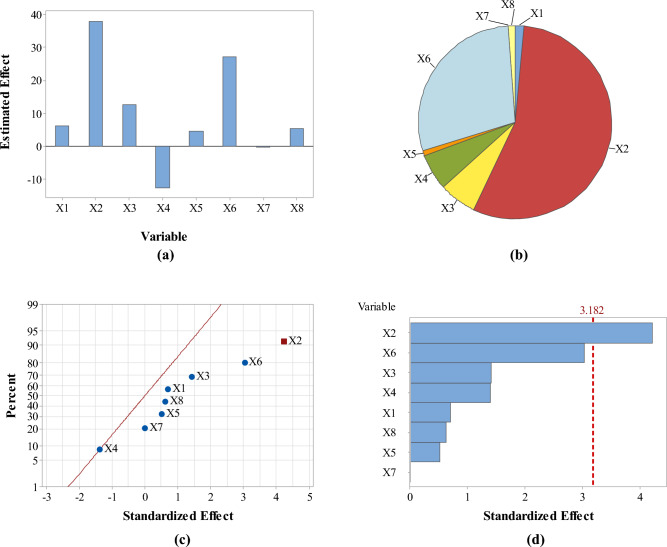
Estimated effects (**a**), contribution % (**b**), normal probability plot of the standardized effects (**c**) and Pareto chart of the standardized effects (**d**) of variables affecting *Citrus* peel-mediated synthesis of SNPs (the fourth response: concentration in ppm). Variables studied are temperature (X1), pH (X2), shaking speed (X3), incubation time (X4), peel extract concentration (X5), AgNO_3_ concentration (X6), extract/AgNO_3_ volume ratio (X7) and dummy factor (X8).

Analysis of responses in terms of the estimated effects and contribution % of variables revealed that pH had the maximum effect on the four responses as compared with the other screened variables (Figs. 3, 4, 5 and 6: a–b). However, pH was directly proportional to A_max_ at λ_400–500_ and concentration of the formed SNPs, while it was inversely proportional to their size and zeta potential. In addition, normal probability plots and Pareto charts of the standardized effects of variables indicated that pH was the only variable with significant effect on the four responses at *p* ≤ 0.05 (Figs. 3, 4, 5 and 6: c–d).

ANOVA and regression results of PBD confirmed the significant effect of pH on *Citrus* peel-mediated synthesis of SNPs. For all the considered responses, the maximum sum of squares (SS), mean square (MS), F-value, regression coefficient (Coef) and normalized regression coefficient (T-value) were recorded for pH. Each of SS and MS indicates the amount of difference in the observed response caused by each variable. Moreover, high F-value indicates that the difference in the response caused by variable is greater than that caused by noise. Regression Coef and T-value represent the contribution of each variable to the variation in response. Furthermore, when *p* value is compared with the risk level (alpha = 0.05), the variable would have a significant effect on the response if the *p*-value is less than alpha. That was the case only when considering pH for all the considered responses (Tables S1–S4).

Therefore and by considering pH (the only significant variable) as the sole predictor for all responses, the regression equations can be shortened as:

$$ {\text{A}}_{{{\text{max}}}} \;{\text{at}}\;\uplambda_{{{4}00 - {5}00}} = {1}.{483} + 0.{739}\;{\text{pH}} + 0.{732}\;{\text{Ct}}\;{\text{Pt}} $$

$$ {\text{Zeta}}\;{\text{size}} = {374}.{2} - {327}.{6}\;{\text{pH}} - {267}\;{\text{Ct}}\;{\text{Pt}} $$

$$ {\text{Zeta}}\;{\text{potential}} = - {13}.{51}0 - {8}.0{73}\;{\text{pH}} - {1}.{69}\;{\text{Ct}}\;{\text{Pt}} $$

$$ {\text{Concentration}} = {31}.{82} + {19}.0{5}\;{\text{pH}} + {36}.0\;{\text{Ct}}\;{\text{Pt}} $$

For the four responses respectively, 1.483, 374.2, − 13.510 and 31.82 are the model intercepts; 0.739, − 327.6, − 8.073 and 19.05 are the regression coefficients of pH; and Ct Pt is the center point response.

Furthermore, model summary for all responses proved that the designed models fit for reasonable description of each response. This was indicated from high values of R-squared (R-Sq) and adjusted R-squared (R-sq(adj)); along with low values of standard error of noise (S). In addition, model summary proved that the designed models can be used to efficiently predict the response for new observations. This was indicated from high values of predicted R-squared (R-sq(pred)) and prediction error sum of squares (PRESS) (Tables S1–S4).

Matching the results obtained herein, it was recorded that pH values of 7–9 are generally optimum for the synthesis of SNPs^27^. The documented optimum alkaline pH range for SNPs synthesis was ascribed to the enhancement of reduction process, quick growth rate, high yield capacity as well as the formation of stable nanoparticles^28^. It was also postulated that alkaline pH permits more hydroxyl groups to be involved in the reduction of silver ions into silver atoms; and permits also more functional groups to be involved in stabilizing the formed SNPs^29^.

After determining the significant variables, further statistical optimization is highly recommended provided that PBD revealed two or more variables with significant effect on SNPs synthesis. In the current study however, only one variable (pH) among the screened ones had significant effect on the four estimated responses. Therefore, no further statistical optimization could be performed. In consequence, it seemed essential to choose only one run that achieved the most enhanced (optimized) SNPs. Based on the results obtained from the runs, run 13, run 2 and run 12 achieved the best characteristics of SNPs (Table 4). Precise consideration of physico-chemical conditions of the three runs revealed that run 2 and run 12 are more economic than run 13 in terms of temperature and the amount of *Citrus* peel biomass required to mediate SNPs synthesis (Table 2). At the same time, run 2 seemed to be even more economic and faster than run 12 in terms of shaking speed and incubation time (Table 2). Thus, run 2 was chosen for the next step involving purification and characterization of the optimized SNPs.

### Purification and characterization of the optimized SNPs

To purify the optimized SNPs, the reaction mixture was centrifuged at high speed then the supernatant containing small nanoparticles was recovered. In addition, cloud point extraction technique allowed further purification of the optimized SNPs. Triton *x*-100 was used as a non-ionic surfactant and capping agent with a molar mass of 647 and cloud point temperature of 70 °C for its critical micellar concentration (1% aqueous solution). Triton *x*-100 can chelate SNPs causing complextaion and entrapment of SNPs in the micelles. Capped SNPs can be then separated in the surfactant-rich phase. Previous studies have pointed out the role of triton x-100 as a stabilizing agent for biogenic SNPs^30^. In this context, triton *x*-100 had a dual reduction and capping role wile synthesizing SNPs that were characterized by spherical shape and average size of 5 nm^31^.

**Figure 7:**
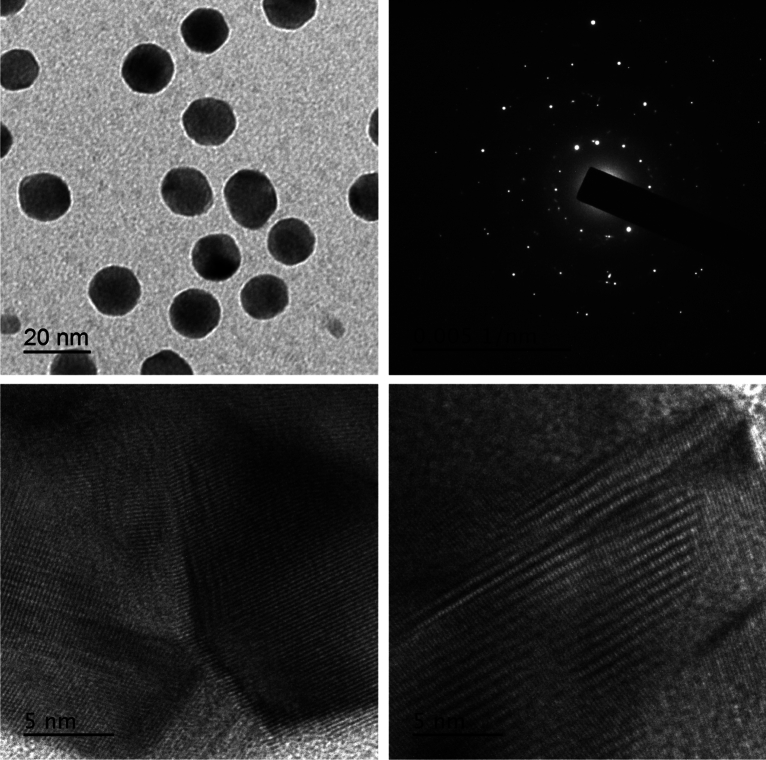
TEM and HR-TEM of optimized SNPs synthesized with the aid of *Citrus* peel extract.

In the current study, TEM characterization of the engineered (optimized and capped) SNPs revealed their typical and highly-regular spherical shape (Fig. 7). The engineered SNPs had much more regular spherical shape as compared with their shape when resulted from the crude synthesis (Fig. 2). HR-TEM micrographs of the engineered SNPs manifested their lattice fringes; with the selected area electron diffraction (SAED) of the engineered SNPs clearly indicated their crystalline nature (Fig. 7).

**Figure 8:**
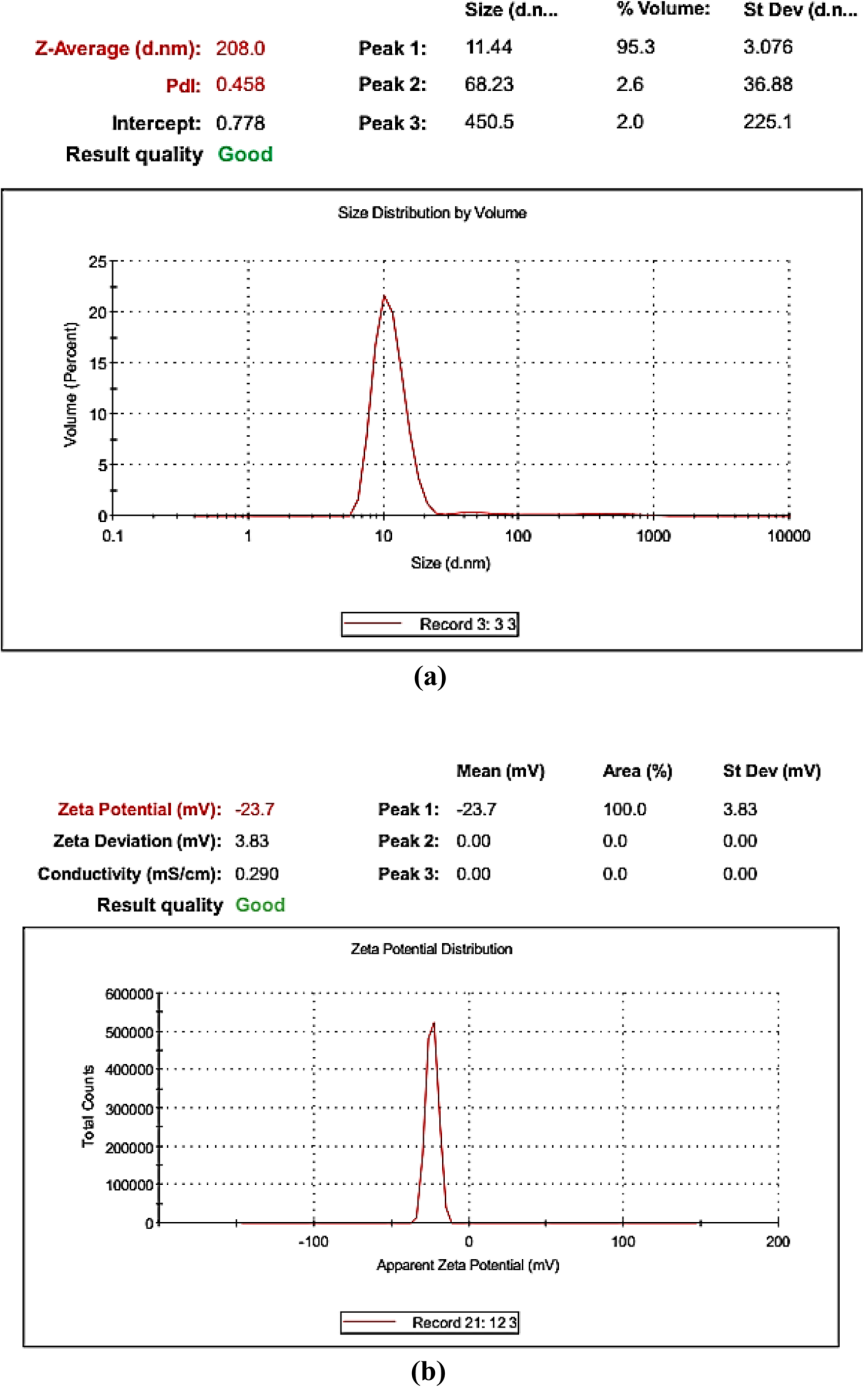
Size distribution (**a**) and zeta potential distribution (**b**) of optimized SNPs synthesized with the aid of *Citrus* peel extract.

Regarding their size, the engineered SNPs had size less than 20 or even 15 nm as revealed from TEM characterization. Such small size was confirmed through zetametry for size analysis where 95.3% of the engineered SNPs had zeta size of 11.44 nm with low standard deviation value of 3.076 nm (Fig. 8a). Zetametry also showed that the engineered SNPs had zeta potential of − 23.7 mV (Fig. 8b) indicating moderate stability in aqueous suspension (stability was further confirmed by following pH change with time as shown in Fig. S1). The features of engineered SNPs are completely different from SNPs initially obtained from crude synthesis (irregular shape, 85 nm average zeta size and − 18.2 mV zeta potential all listed in Table 4 for the center point run). This difference indicates how well the engineering protocol followed in the current study could enhance the properties of SNPs. Furthermore, the average number of atoms per each nanoparticle was calculated; and it was found to be 45,934 atoms per nanoparticle. Such low number of atoms per nanoparticle may result from the small size of the engineered SNPs.

### Antimicrobial activity of SNPs

**Figure 9:**
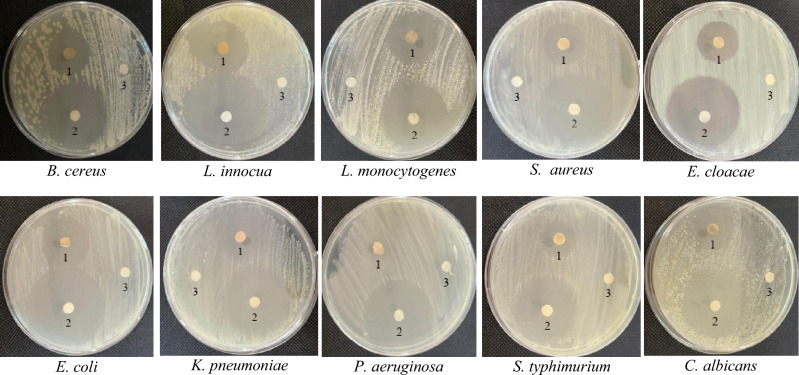
Disk diffusion test of SNPs (1), gentamicin (2) and distilled water (3) against different microbial pathogens.

**Figure 10:**
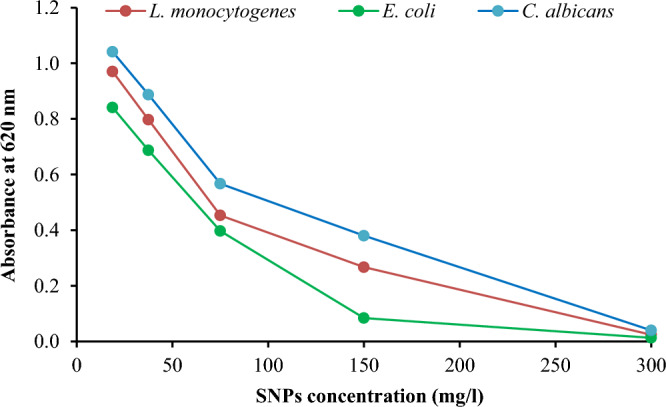
Absorbance of liquid cultures of different microbial pathogens as affected by serial dilutions of SNPs.

Following disk diffusion method, the engineered SNPs showed marked antimicrobial activity against all the tested microbes; with the maximum inhibition zone diameter recorded for* E*.* coli* followed by *L*.* monocytogenes* and *C*. *albicans* (Fig. 9 and Table 5). Therefore, MIC and MBC of SNPs were detected for these microbes. For the three microbes, MIC value of SNPs was found to be 300 mg/l, while MBC value was found to be 300 mg/l for *E*.* coli* and 400 mg/l for each of *L*.* monocytogenes* and *C*. *albicans* (Fig. 10 and Table 6). This indicates the marked antimicrobial activity of SNPs synthesized herein using *Citrus* peel aqueous extract as compared with the activity of SNPs recorded elsewhere. In this context, SNPs synthesized using *Lysiloma acapulcensis* stem and root aqueous extract exhibited antimicrobial activity with inhibition zone diameter reaching 16 mm for *S. aureus*, 18 mm for *E. coli* and 15 mm for *P. aeruginosa*^32^. In addition, SNPs could inhibit the growth of *E. coli* with inhibition zone diameter of 14.5 mm and MIC value of 300 mg/l^33^. Also, SNPs synthesized using leaf aqueous extracts of *Crossopteryx febrifuga*, *Brillantaisia patula* and *Senna siamea* exerted antibacterial activity against *S. aureus*, *E. coli* and *P. aeruginosa*^34^.

**Table 5:** Diameter of clear zone formed by the enhanced SNPs and gentamicin against different microbial pathogens (values listed are mean of three replica ± standard deviation with different letters referring to significant difference at *p* ≤ 0.05).

Pathogen	Diameter of clear zone (mm)	
	SNP	Gentamicin
*Bacillus cereus*	24.5 d ± 0.6	36.7 d ± 1.0
*Listeria innocua*	29.7 c ± 0.6	45.5 a ± 1.6
*Listeria monocytogenes*	31.8 b ± 0.6	36.0 d ± 0.6
*Staphylococcus aureus*	24.5 d ± 0.6	40.6 c ± 0.6
*Enterobacter cloacae*	24.6 d ± 0.0	39.6 c ± 0.6
*Escherichia coli*	33.2 a ± 0.6	43.4 b ± 1.2
*Klebsiella pneumoniae*	30.0 c ± 0.6	39.1 c ± 1.1
*Pseudomonas aeruginosa*	11.1 f. ± 1.0	34.1 e ± 0.6
*Salmonella typhimurium*	23.2 e ± 0.6	35.5 de ± 0.6
*Candida albicans*	31.7 b ± 0.6	40.3 c ± 0.6
Least significant difference	1.1	1.6

**Table 6:** MBC and MIC of SNPs against different microbial pathogens and the tolerance level of these microbes.

Pathogen	MBC (mg/l)	MIC (mg/l)	Tolerance level
*Listeria monocytogenes*	400	300	1.33
*Escherichia coli*	300	300	1.00
*Candida albicans*	400	300	1.33

Interestingly, MBC/MIC ratio (tolerance level or TL) calculated herein indicated that *E. coli* had TL value of 1, while each of *L. monocytogenes* and *C. albicans* had TL value of 1.33 (Table 6). In this regard, TL was recorded as a parameter indicating antimicrobial capacity of the concerned material. An antimicrobial agent is bacteriostatic when TL value of the tested microbe ≥ 16. Meanwhile, TL value ≤ 4 indicates that the antimicrobial agent is bactericidal^21^. Bacteriostatic agents prevent or inhibit the growth of microbes by keeping them in the stationary phase of growth, while bactericidal agents kill the microbes. Therefore, the biogenic SNPs considered in the current study can be considered as bactericidal against the three tested microbes since their TL value is less than 4. Similar results about TL of some microbes towards biogenic SNPs were recently recorded^35,36^.

**Figure 11:**
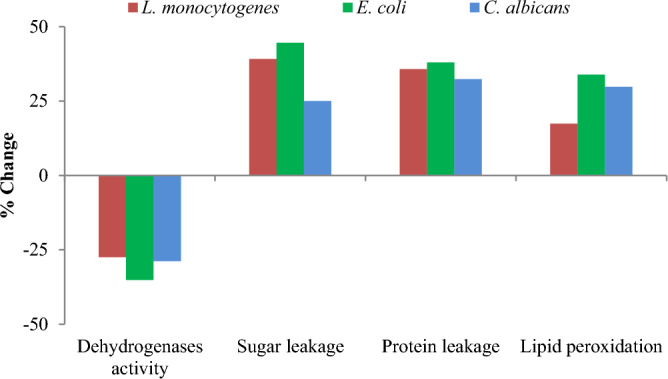
Activity of respiratory chain dehydrogenases, cellular sugar leakage, cellular protein leakage and lipid peroxidation of different microbial pathogens as affected by SNPs.

**Figure 12:**
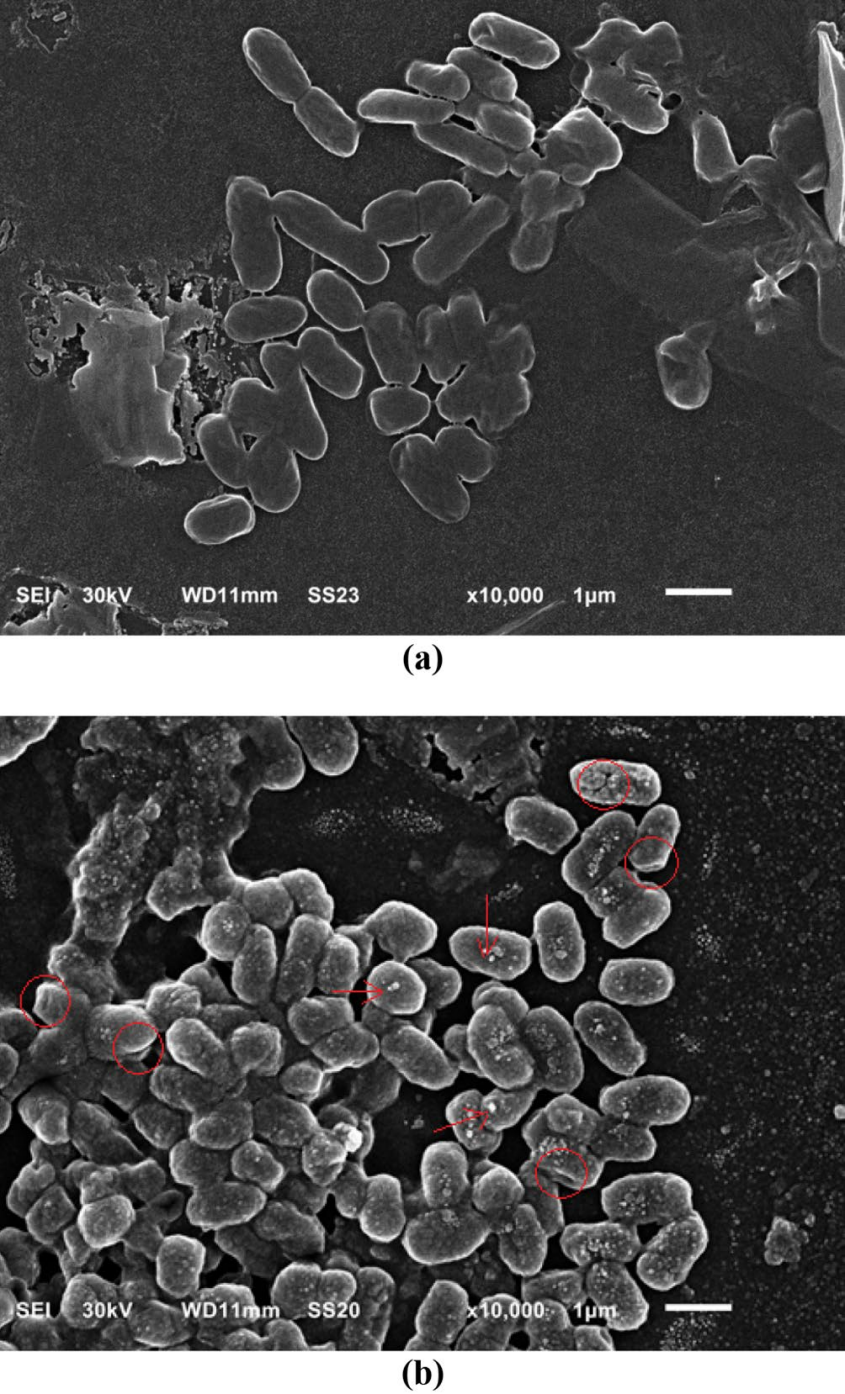
Scanning electron micrographs of SNPs-untreated (**a**) and treated (**b**) cells of *E. coli*. Red circles refer to pores and/or invaginations in the microbial cells especially at the cell poles. Circles also refer to cell destruction and/or distortion. Meanwhile, red arrows refer to accumulation of SNPs on the microbial cells (magnification of 10,000x).

**Figure 13:**
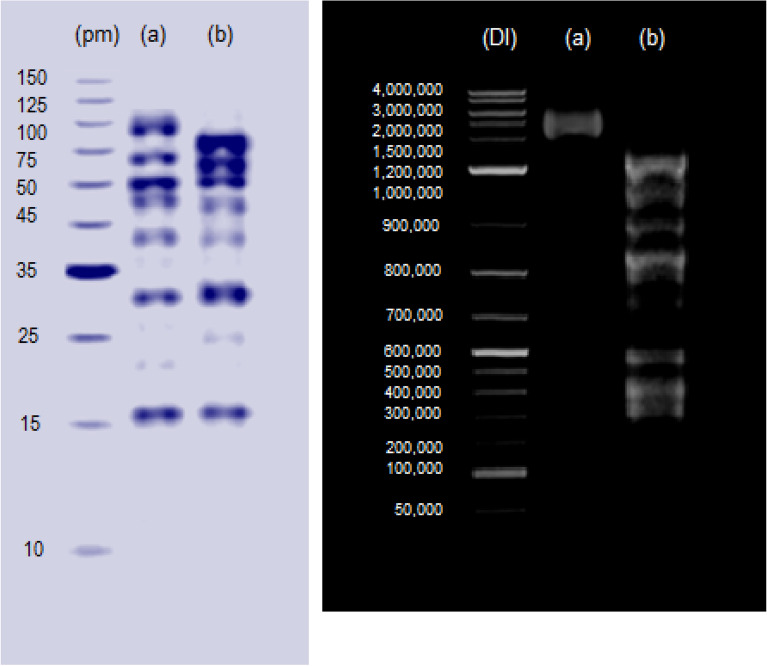
Protein patterning (left side with pm = protein marker) and DNA fragmentation (right side with Dl = DNA ladder) of SNPs-treated (**a**) and untreated (**b**) cells of *E. coli*.

To uncover the mechanism of SNPs antimicrobial activity against the microbes concerned herein, some biochemical features of the microbial cells were studied. Results obtained showed that the engineered SNPs could inhibit respiratory chain dehydrogenases of *E*.* coli*,* L*.* monocytogenes* and *C*. *albicans*; with marked increase in cellular leakage of sugars and proteins; and increased extent of lipid peroxidation. However, the maximum % change in these parameters was recorded for *E*.* coli* when compared with* L*.* monocytogenes* and *C*. *albicans* (Fig. 11). Thus, *E*.* coli* was subjected to further analysis by scanning electron microscopy (SEM) and molecular studies of proteins and DNA. Treating *E*.* coli* with SNPs caused marked alterations in cell ultrastructure as revealed by SEM. SNPs-treated cells had clear pores and/or invaginations especially at the cell poles. In addition, SNPs treatment resulted in general distortion and/or destruction of microbial cells. Furthermore, accumulation of SNPs on the microbial cells could be indicted via SEM micrographs (Fig. 12). Also, treating *E. coli* with SNPs resulted in the appearance of some new protein bands with molecular weight of 116.475, 107.672, 70.926, 47.91, 44.313 and 20.461 kDa. On contrary, SNPs treatment resulted in the disappearance of some original protein bands with molecular weight of 77.893, 62.688, 54.686, 46.312, 33.558 and 24.438 kDa. In addition, some protein bands were almost common between the treated and untreated microbe. The largest common band was of molecular weight of 92.004 kDa, while the smallest common band was of molecular weight of 15.237 kDa (Fig. 13 and Tables S5–S7). For DNA, treating *E. coli* with SNPs resulted in the appearance of some new DNA fragments with size of 1726.362, 1466.109 and 1259.550 base pair. Meanwhile, SNPs treatment resulted in disappearance of the original DNA fragments; and no DNA fragments were common between the treated and untreated microbe (Fig. 13 and Tables S8–S10).

In this context, microbial cell membrane was identified as the first target of damage caused by SNPs. Previous studies proved that SNPs could interfere with microbial cell membrane and its transport system^33^. In addition, antimicrobial activity of SNPs could be ascribed to their induction of oxidative stress by stimulating overproduction of free radicals and active oxygen species. These highly reactive by-products could hit and consequently disrupt cellular membrane, organelles and biomolecules. Therefore, the increased lipid peroxidation along with cellular leakage of sugars and proteins as well as ultrastructural malformation recorded herein for SNPs-treated *E. coli* can be attributed to the oxidative stress induced by SNPs. Similar findings were reported in other studies. For instance, biogenic SNPs exerted potent antimicrobial activity against *C. albicans* by stimulating lipid peroxidation with consequent leakage of cellular sugars and proteins; as well as ultrastructural changes of the treated microbe^37^. Also, deactivation of respiratory dehydrogenases recorded herein for SNPs-treated *E. coli* comes in parallel with the inhibition of such enzymes as previously recorded in other microbes^38^. This effect could be also linked with oxidative stress resulting from nanoparticles treatment. Moreover, the disappearance of certain proteins and DNA fragments as reported in the current study for SNPs-treated *E. coli* can be explained based on the damage of these macromolecules or their leakage from the microbial cells as a result of SNPs treatment. Furthermore, it was postulated that SNPs could intercalate with nitrogen bases of DNA causing the hydrogen bonds between the two strands to weaken with consequent DNA damage^33^. However, appearance of some new proteins and DNA fragments can be considered as a trial from the stressed bacteria to withstand stress originating from SNPs treatment. The ability of biogenic SNPs to target protein and DNA damage was recently proven^39,40^.

## Conclusion

Results of the current study indicate that aqueous extract of *Citrus* peel can be simply used for green synthesis of SNPs. Among the factors affecting properties and stability of SNPs, pH is the most important. The as-prepared SNPs possess potent antimicrobial action. So, these biogenic SNPs can be used as bactericidal agent especially against *E. coli*. However, it is worthy to note that one of the main challenges when using SNPs as antimicrobial agents is its possible cytotoxicity against human cells. Although some studies pointed out to the safe behavior of SNPs on human cells, assay of SNPs cytotoxicity may be necessary to confirm such effect. Further investigation is thus needed to make the conclusion and recommendation of the current study more robust. Otherwise, the biogenic SNPs can be employed as antimicrobial agents to sterilize tools, fabrics, surfaces and other stuff away from being directly used as antimicrobial drugs.

### Supplementary Information

Supplementary Information.

## Data Availability

All data used in this study are included in the article and its supplementary file.
