# Supplementary material for: Plackett–Burman screening of physico-chemical variables affecting Citrus peel-mediated synthesis of silver nanoparticles and their antimicrobial activity
Source: Sci Rep. 2024 Apr 6;14:8079. doi: 10.1038/s41598-024-58102-x (PMC10998881; doi:10.1038/s41598-024-58102-x)
Supplement: Supplementary file 1 — Supplementary Information. [file 41598_2024_58102_MOESM1_ESM.docx]

**Table S1.** Analysis of variance (ANOVA), coded coefficients (regression), model summary and regression equation of Plackett-Burman design used to screen variables affecting *Citrus* peel-mediated synthesis of SNPs (the first response: A_max_ at λ_400-500_). Variables studied are temperature (X1), pH (X2), shaking speed (X3), incubation time (X4), peel extract concentration (X5), AgNO_3_ concentration (X6), extract/AgNO_3_ volume ratio (X7) and dummy factor (X8).

**A B**

| Source | Df | SS | MS | F-value | *p*-value | T-value | Coef | SE coef |
| --- | --- | --- | --- | --- | --- | --- | --- | --- |
| Model | 9 | 9.8322 | 1.0925 | 1.82 | 0.339 |  |  |  |
| Linear | 8 | 9.3381 | 1.1673 | 1.94 | 0.317 |  |  |  |
| X1 | 1 | 0.4481 | 0.4481 | 0.75 | 0.451 | 0.86 | 0.193 | 0.224 |
| X2 | 1 | 6.5461 | 6.5461 | 10.89 | 0.046 | 3.30 | 0.739 | 0.224 |
| X3 | 1 | 0.0431 | 0.0431 | 0.07 | 0.806 | 0.27 | 0.060 | 0.224 |
| X4 | 1 | 0.1402 | 0.1402 | 0.23 | 0.662 | -0.48 | -0.108 | 0.224 |
| X5 | 1 | 1.6628 | 1.6628 | 2.77 | 0.195 | 1.66 | 0.372 | 0.224 |
| X6 | 1 | 0.1580 | 0.1580 | 0.26 | 0.644 | 0.51 | 0.115 | 0.224 |
| X7 | 1 | 0.3397 | 0.3397 | 0.57 | 0.507 | 0.75 | 0.168 | 0.224 |
| X8 | 1 | 0.0001 | 0.0001 | 0.00 | 0.989 | -0.01 | -0.003 | 0.224 |
| Curvature | 1 | 0.4940 | 0.4940 | 0.82 | 0.431 |  |  |  |
| Error | 3 | 1.8033 | 0.6011 |  |  |  |  |  |
| Total | 12 | 11.636 |  |  |  |  |  |  |

Model summary:

| S | R-sq | R-sq(adj) | PRESS | R-sq(pred) |
| --- | --- | --- | --- | --- |
| 0.775307 | 84.50% | 38.01% | * | ** |

Regression equation:

A_max_ at λ_400-500_ = 1.483 + 0.193 X1 + 0.739 X2 + 0.060 X3 - 0.108 X4 + 0.372 X5 + 0.115 X6 + 0.168 X7 - 0.003 X8 + 0.732 Ct Pt

**Table S2.** Analysis of variance (ANOVA), coded coefficients (regression), model summary and regression equation of Plackett-Burman design used to screen variables affecting *Citrus* peel-mediated synthesis of SNPs (the second response: zeta size in nm). Variables studied are temperature (X1), pH (X2), shaking speed (X3), incubation time (X4), peel extract concentration (X5), AgNO_3_ concentration (X6), extract/AgNO_3_ volume ratio (X7) and dummy factor (X8).

| Source | Df | SS | MS | F-value | *p*-value | T-value | Coef | SE coef |
| --- | --- | --- | --- | --- | --- | --- | --- | --- |
| Model | 9 | 1424219 | 158247 | 11.86 | 0.033 |  |  |  |
| Linear | 8 | 1358189 | 169774 | 12.73 | 0.030 |  |  |  |
| X1 | 1 | 24630 | 24630 | 1.85 | 0.267 | -1.36 | -45.3 | 33.3 |
| X2 | 1 | 1288208 | 1288208 | 96.55 | 0.002 | -9.83 | -327.6 | 33.3 |
| X3 | 1 | 766 | 766 | 0.06 | 0.826 | -0.24 | -8.0 | 33.3 |
| X4 | 1 | 10932 | 10932 | 0.82 | 0.432 | 0.91 | 30.2 | 33.3 |
| X5 | 1 | 185 | 185 | 0.01 | 0.914 | 0.12 | 3.9 | 33.3 |
| X6 | 1 | 2171 | 2171 | 0.16 | 0.714 | -0.40 | -13.4 | 33.3 |
| X7 | 1 | 11234 | 11234 | 0.84 | 0.426 | -0.92 | -30.6 | 33.3 |
| X8 | 1 | 20063 | 20063 | 1.50 | 0.308 | 1.23 | 40.9 | 33.3 |
| Curvature | 1 | 66030 | 66030 | 4.95 | 0.113 |  |  |  |
| Error | 3 | 40025 | 13342 |  |  |  |  |  |
| Total | 12 | 1464244 |  |  |  |  |  |  |

Model summary:

| S | R-sq | R-sq(adj) | PRESS | R-sq(pred) |
| --- | --- | --- | --- | --- |
| 115.506 | 97.27% | 89.07% | * | ** |

Regression equation:

Zeta size = 374.2 - 45.3 X1 - 327.6 X2 - 8.0 X3 + 30.2 X4 + 3.9 X5 - 13.4 X6 - 30.6 X7 + 40.9 X8 - 267 Ct Pt

**Table S3.** Analysis of variance (ANOVA), coded coefficients (regression), model summary and regression equation of Plackett-Burman design used to screen variables affecting *Citrus* peel-mediated synthesis of SNPs (the third response: zeta potential in mV). Variables studied are temperature (X1), pH (X2), shaking speed (X3), incubation time (X4), peel extract concentration (X5), AgNO_3_ concentration (X6), extract/AgNO_3_ volume ratio (X7) and dummy factor (X8).

| Source | Df | SS | MS | F-value | *p*-value | T-value | Coef | SE coef |
| --- | --- | --- | --- | --- | --- | --- | --- | --- |
| Model | 9 | 856.5 | 95.2 | 18.93 | 0.017 |  |  |  |
| Linear | 8 | 853.9 | 106.7 | 21.23 | 0.015 |  |  |  |
| X1 | 1 | 0.1 | 0.1 | 0.02 | 0.894 | -0.14 | -0.093 | 0.647 |
| X2 | 1 | 782.1 | 782.1 | 155.56 | 0.001 | -12.47 | -8.073 | 0.647 |
| X3 | 1 | 43.5 | 43.5 | 8.65 | 0.060 | 2.94 | 1.903 | 0.647 |
| X4 | 1 | 0.1 | 0.1 | 0.03 | 0.880 | -0.16 | -0.107 | 0.647 |
| X5 | 1 | 12.6 | 12.6 | 2.52 | 0.211 | 1.59 | 1.027 | 0.647 |
| X6 | 1 | 2.1 | 2.1 | 0.41 | 0.569 | 0.64 | 0.413 | 0.647 |
| X7 | 1 | 6.8 | 6.8 | 1.35 | 0.329 | 1.16 | 0.753 | 0.647 |
| X8 | 1 | 6.5 | 6.5 | 1.30 | 0.338 | -1.14 | -0.737 | 0.647 |
| Curvature | 1 | 2.6 | 2.6 | 0.52 | 0.521 |  |  |  |
| Error | 3 | 15.1 | 5.0 |  |  |  |  |  |
| Total | 12 | 871.6 |  |  |  |  |  |  |

Model summary:

| S | R-sq | R-sq(adj) | PRESS | R-sq(pred) |
| --- | --- | --- | --- | --- |
| 2.24227 | 98.27% | 93.08% | * | *** |

Regression equation:

Zeta potential = -13.510 - 0.093 X1 - 8.073 X2 + 1.903 X3 - 0.107 X4 + 1.027 X5 + 0.413 X6 + 0.753 X7 - 0.737 X8 - 1.69 Ct Pt

**Table S4.** Analysis of variance (ANOVA), coded coefficients (regression), model summary and regression equation of Plackett-Burman design used to screen variables affecting *Citrus* peel-mediated synthesis of SNPs (the fourth response: concentration in ppm). Variables studied are temperature (X1), pH (X2), shaking speed (X3), incubation time (X4), peel extract concentration (X5), AgNO_3_ concentration (X6), extract/AgNO_3_ volume ratio (X7) and dummy factor (X8).

| Source | Df | SS | MS | F-value | *p*-value | T-value | Coef | SE coef |
| --- | --- | --- | --- | --- | --- | --- | --- | --- |
| Model | 9 | 9031.6 | 1003.5 | 4.09 | 0.137 |  |  |  |
| Linear | 8 | 7838.3 | 979.8 | 3.99 | 0.141 |  |  |  |
| X1 | 1 | 117.9 | 117.9 | 0.48 | 0.538 | 0.69 | 3.13 | 4.52 |
| X2 | 1 | 4356.4 | 4356.4 | 17.75 | 0.024 | 4.21 | 19.05 | 4.52 |
| X3 | 1 | 488.5 | 488.5 | 1.99 | 0.253 | 1.41 | 6.38 | 4.52 |
| X4 | 1 | 477.0 | 477.0 | 1.94 | 0.258 | -1.39 | -6.31 | 4.52 |
| X5 | 1 | 64.6 | 64.6 | 0.26 | 0.643 | 0.51 | 2.32 | 4.52 |
| X6 | 1 | 2240.2 | 2240.2 | 9.13 | 0.057 | 3.02 | 13.66 | 4.52 |
| X7 | 1 | 0.0 | 0.0 | 0.00 | 0.995 | -0.01 | -0.03 | 4.52 |
| X8 | 1 | 93.6 | 93.6 | 0.38 | 0.581 | 0.62 | 2.79 | 4.52 |
| Curvature | 1 | 1193.3 | 1193.3 | 4.86 | 0.115 |  |  |  |
| Error | 3 | 736.3 | 245.4 |  |  |  |  |  |
| Total | 12 | 9767.9 |  |  |  |  |  |  |

Model summary:

| S | R-sq | R-sq(adj) | PRESS | R-sq(pred) |
| --- | --- | --- | --- | --- |
| 15.6664 | 92.46% | 69.85% | * | ** |

Regression equation:

Concentration = 31.82 + 3.13 X1 + 19.05 X2 + 6.38 X3 - 6.31 X4 + 2.32 X5 + 13.66 X6 - 0.03 X7 + 2.79 X8 + 36.0 Ct Pt

**Fig. S1.** pH-indicated stability of the engineered SNPs synthesized with the aid of *Citrus* peel extract


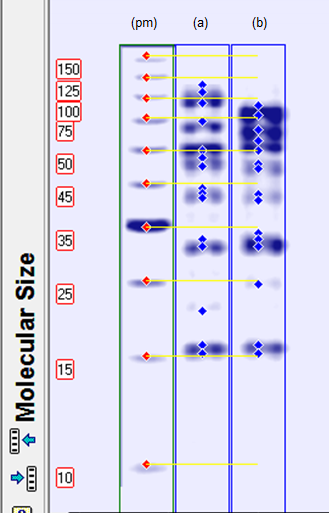


**Fig. S2.** Computerized detection and molecular weight calculation for protein fingerprinting pattern of SNPs-treated (a) and untreated (b) cells of *E. coli*; with protein marker (pm)


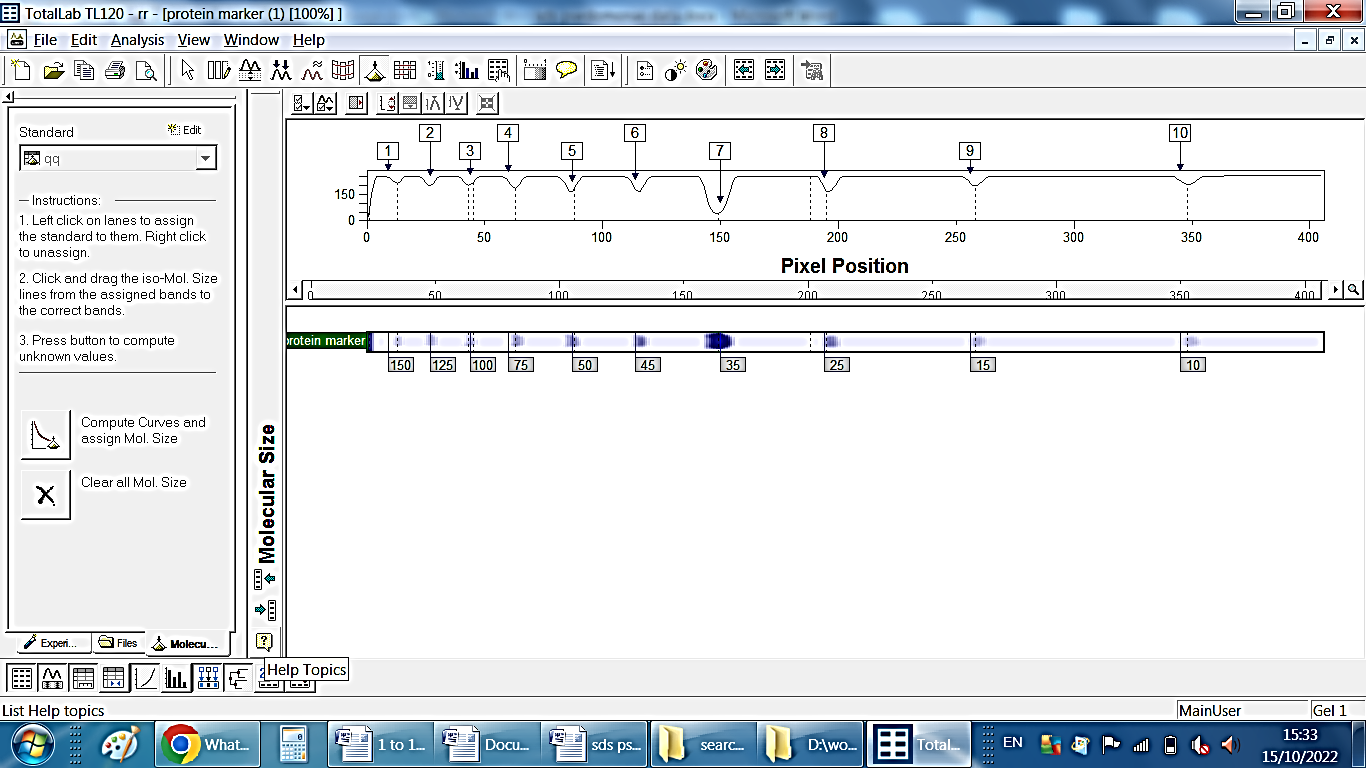


**(pm)**


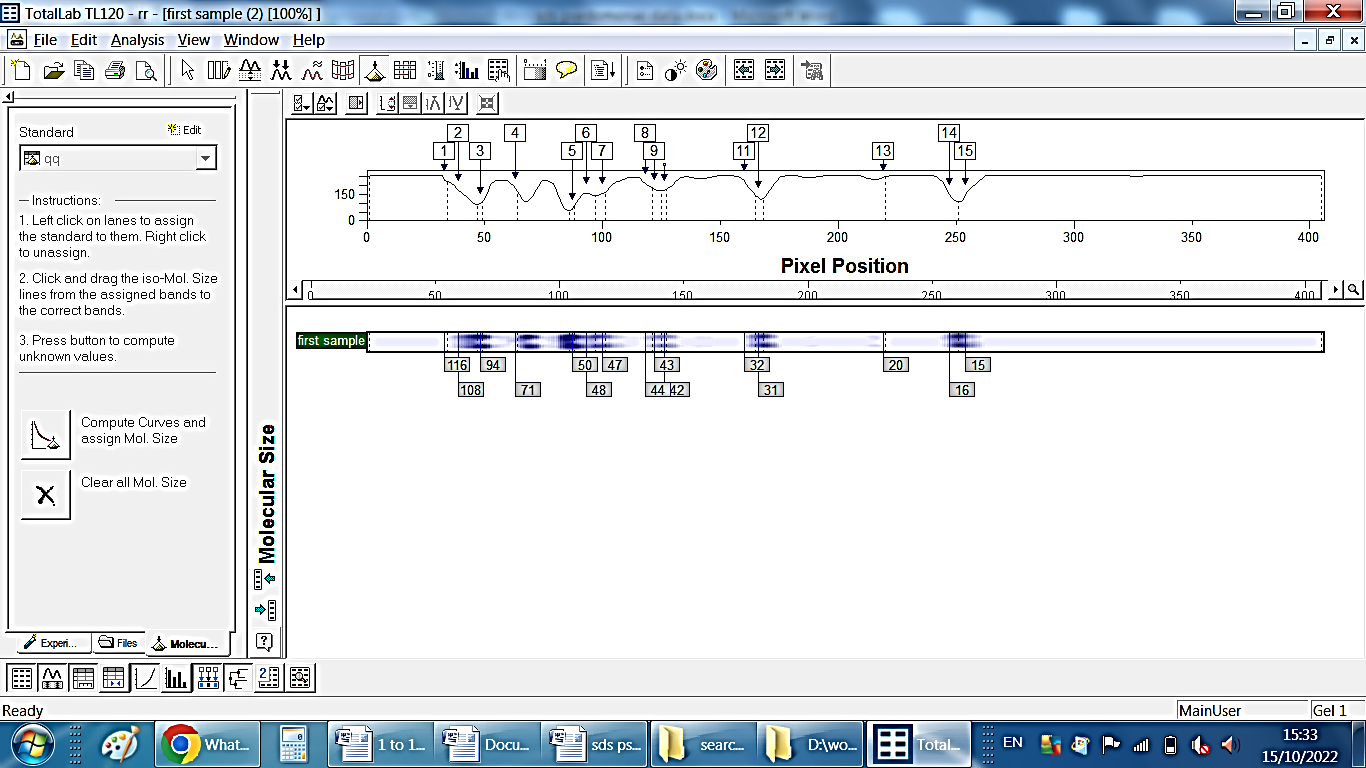


**(a)**


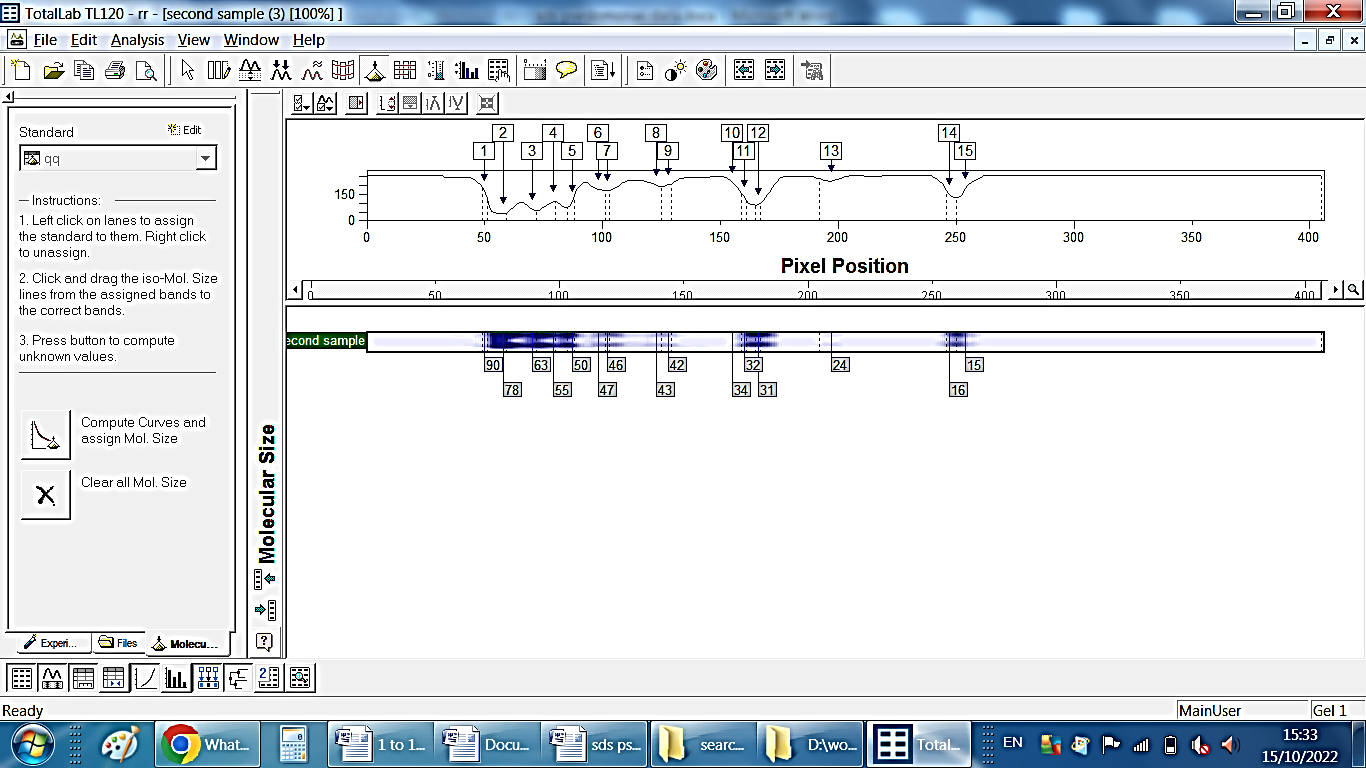


**(b)**

**Fig. S3.** Data analysis of protein pattern of SNPs-treated (a) and untreated (b) cells of *E. coli*; with protein marker (pm)

**Table S5.** Data analysis of protein pattern of SNPs-treated (a) and untreated (b) cells of *E. coli*; with protein marker (pm)

| (pm) | | | (a) | | | (b) | | |
| --- | --- | --- | --- | --- | --- | --- | --- | --- |
| Band  No. | Lane  % | Molecular weight (kDa) | Band  No. | Lane  % | Molecular weight (kDa) | Band  No. | Lane  % | Molecular weight (kDa) |
| 1 | 2.71 | 150 | 1 | 9.1 | 116.475 | 1 | 0.4 | 90.392 |
| 2 | 7.38 | 125 | 2 | 2.25 | 107.672 | 2 | 0.53 | 77.893 |
| 3 | 0.42 | 100 | 3 | 0.21 | 93.616 | 3 | 1.13 | 62.688 |
| 4 | 4.46 | 75 | 4 | 3.22 | 70.926 | 4 | 0.73 | 54.686 |
| 5 | 5.98 | 50 | 5 | 0.13 | 50 | 5 | 0.3 | 50 |
| 6 | 14.24 | 45 | 6 | 1.32 | 47.91 | 6 | 2.74 | 46.861 |
| 7 | 9.08 | 35 | 7 | 0.64 | 46.563 | 7 | 0.38 | 46.312 |
| 8 | 1.71 | 25 | 8 | 4.73 | 44.313 | 8 | 5.12 | 43.204 |
| 9 | 15.86 | 15 | 9 | 0.77 | 43.445 | 9 | 0.87 | 41.873 |
| 10 | 23.1 | 10 | 10 | 0.37 | 42.428 | 10 | 7.82 | 33.558 |
|  |  |  | 11 | 9.61 | 32.223 | 11 | 0.32 | 32.223 |
|  |  |  | 12 | 0.43 | 30.746 | 12 | 0.2 | 30.746 |
|  |  |  | 13 | 13.48 | 20.461 | 13 | 14.74 | 24.438 |
|  |  |  | 14 | 7.81 | 16.127 | 14 | 0.65 | 16.127 |
|  |  |  | 15 | 41.66 | 15.237 | 15 | 43.16 | 15.237 |

**Table S6.** Variation in protein bands of SNPs-treated (a) and untreated (b) cells of *E. coli*

| Ref. Band | (a) | | | (b) | | |
| --- | --- | --- | --- | --- | --- | --- |
|  | Band No. | Lane % | Molecular weight (kDa) | Band No. | Lane % | Molecular weight (kDa) |
| Band 1 | 1 | 9.1 | 116.475 |  |  |  |
| Band 2 | 2 | 2.25 | 107.672 |  |  |  |
| Band 3 | 3 | 0.21 | 93.616 | 1 | 0.4 | 90.392 |
| Band 4 |  |  |  | 2 | 0.53 | 77.893 |
| Band 5 | 4 | 3.22 | 70.926 |  |  |  |
| Band 6 |  |  |  | 3 | 1.13 | 62.688 |
| Band 7 |  |  |  | 4 | 0.73 | 54.686 |
| Band 8 | 5 | 0.13 | 50 | 5 | 0.3 | 50 |
| Band 9 | 6 | 1.32 | 47.91 |  |  |  |
| Band 10 | 7 | 0.64 | 46.563 | 6 | 2.74 | 46.861 |
| Band 11 |  |  |  | 7 | 0.38 | 46.312 |
| Band 12 | 8 | 4.73 | 44.313 |  |  |  |
| Band 13 | 9 | 0.77 | 43.445 | 8 | 5.12 | 43.204 |
| Band 14 | 10 | 0.37 | 42.428 | 9 | 0.87 | 41.873 |
| Band 15 |  |  |  | 10 | 7.82 | 33.558 |
| Band 16 | 11 | 9.61 | 32.223 | 11 | 0.32 | 32.223 |
| Band 17 | 12 | 0.43 | 30.746 | 12 | 0.2 | 30.746 |
| Band 18 |  |  |  | 13 | 14.74 | 24.438 |
| Band 19 | 13 | 13.48 | 20.461 |  |  |  |
| Band 20 | 14 | 7.81 | 16.127 | 14 | 0.65 | 16.127 |
| Band 21 | 15 | 41.66 | 15.237 | 15 | 43.16 | 15.237 |

**Table S7.** Matrix data of protein pattern parameters of SNPs-treated (a) and untreated (b) cells of *E. coli*

| Ref. Band | Molecular weight (kDa) | (a) | (b) |
| --- | --- | --- | --- |
| Band 1 | 116.475 | 1 | 0 |
| Band 2 | 107.672 | 1 | 0 |
| Band 3 | 92.004 | 1 | 1 |
| Band 4 | 77.893 | 0 | 1 |
| Band 5 | 70.926 | 1 | 0 |
| Band 6 | 62.688 | 0 | 1 |
| Band 7 | 54.686 | 0 | 1 |
| Band 8 | 50 | 1 | 1 |
| Band 9 | 47.91 | 1 | 0 |
| Band 10 | 46.712 | 1 | 1 |
| Band 11 | 46.312 | 0 | 1 |
| Band 12 | 44.313 | 1 | 0 |
| Band 13 | 43.324 | 1 | 1 |
| Band 14 | 42.15 | 1 | 1 |
| Band 15 | 33.558 | 0 | 1 |
| Band 16 | 32.223 | 1 | 1 |
| Band 17 | 30.746 | 1 | 1 |
| Band 18 | 24.438 | 0 | 1 |
| Band 19 | 20.461 | 1 | 0 |
| Band 20 | 16.127 | 1 | 1 |
| Band 21 | 15.237 | 1 | 1 |


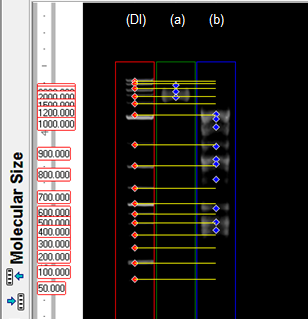


**Fig. S4.** Computerized detection and length calculation for fragmented DNA pattern of SNPs-treated (a) and untreated (b) cells of *E. coli*; with DNA ladder (Dl)

**
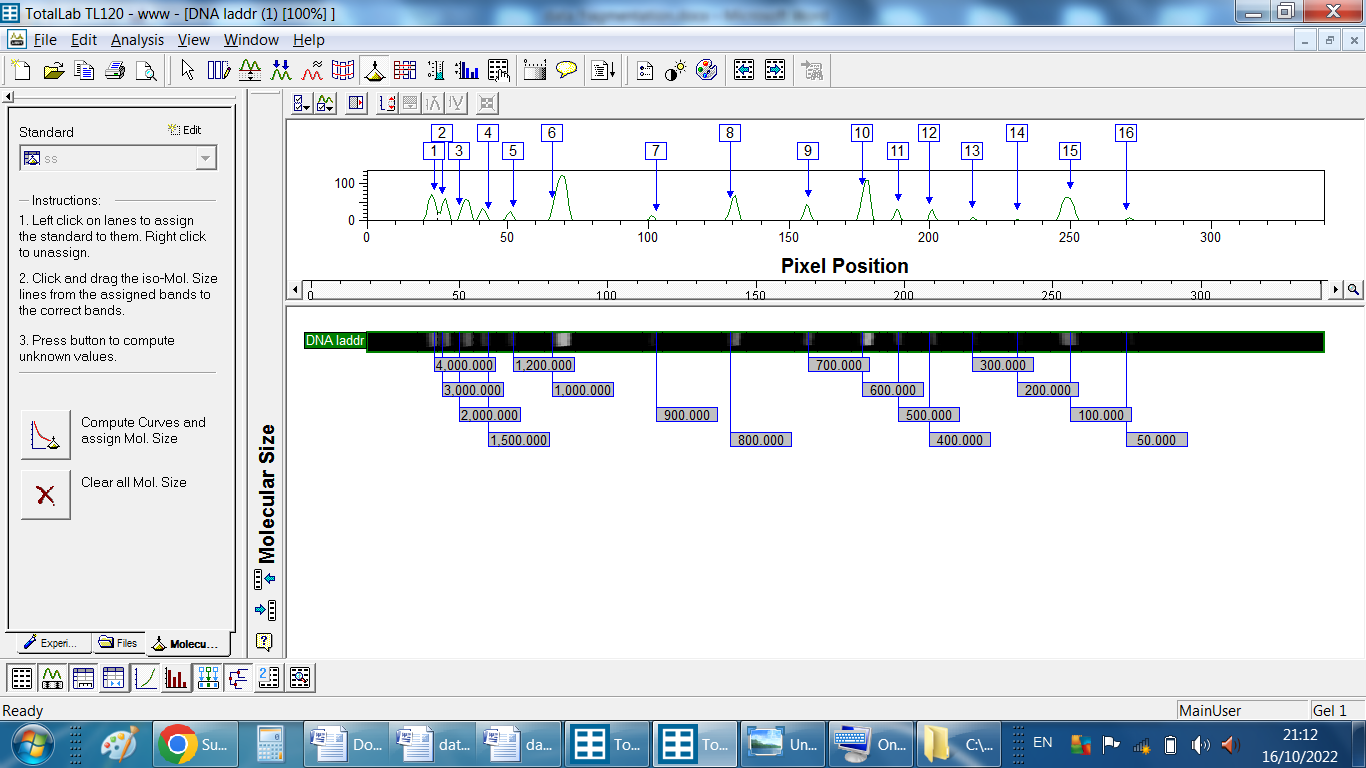
**

**(Dl)**

**
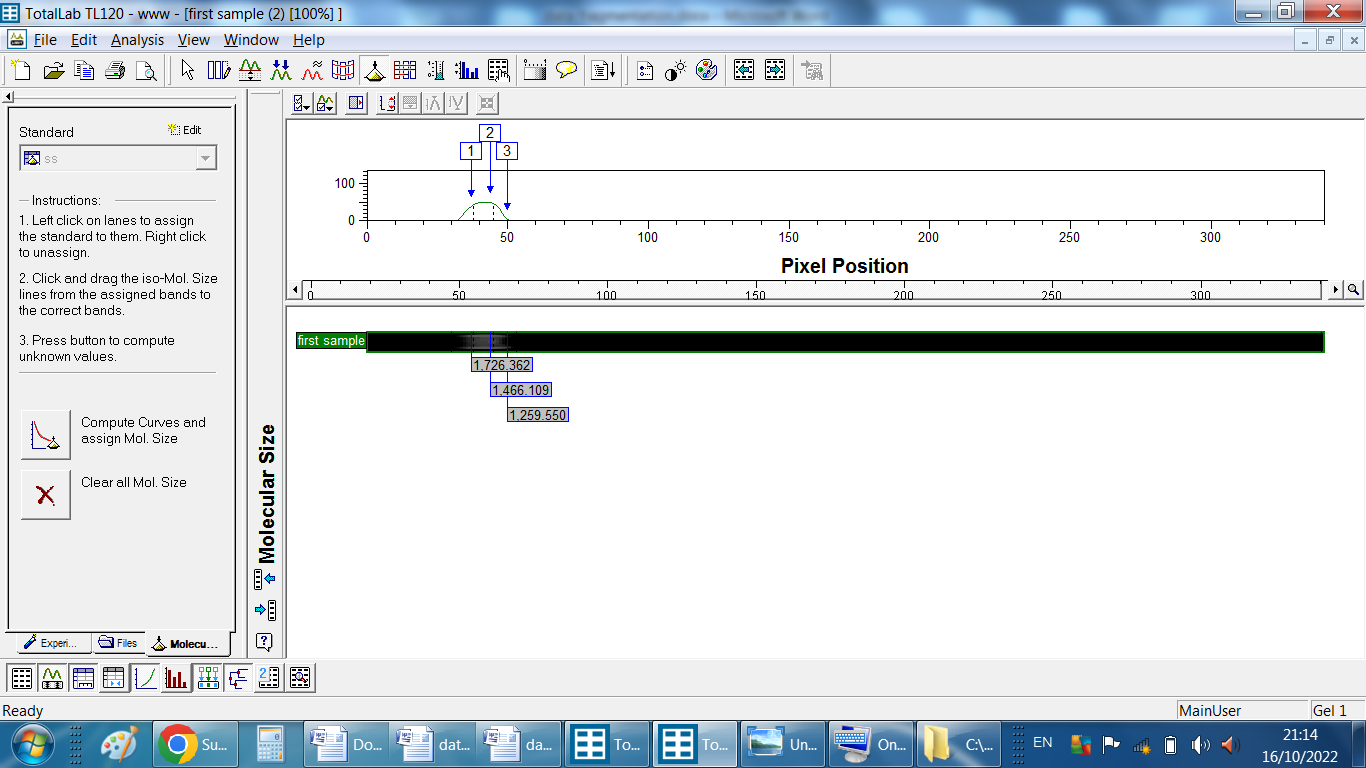
**

**(a)**

**
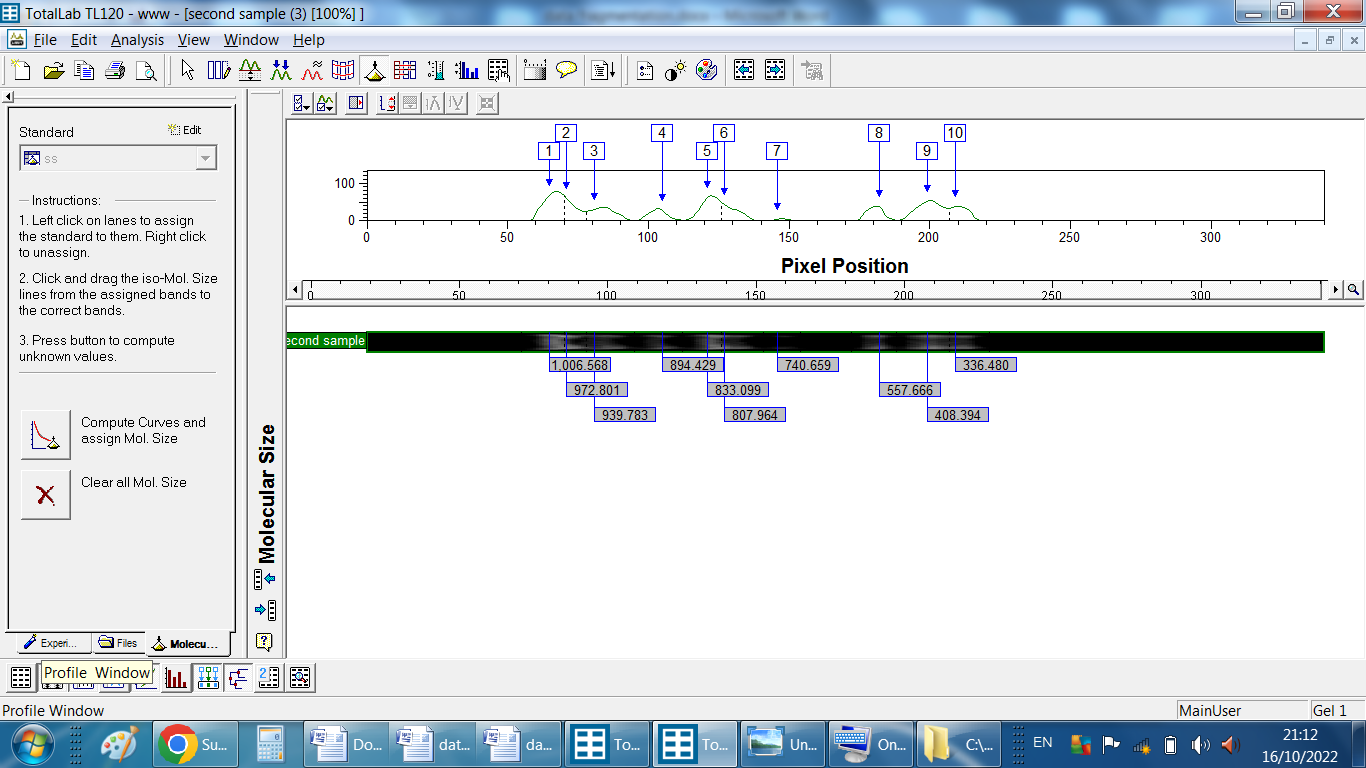
**

**(b)**

**Fig. S5.** Data analysis of fragmented DNA pattern of SNPs-treated (a) and untreated (b) cells of *E. coli*; with DNA ladder (Dl)

**Table S8.** Data analysis of fragmented DNA pattern of SNPs-treated (a) and untreated (b) cells of *E. coli*; with DNA ladder (Dl)

| (Dl) | | | (a) | | | (b) | | |
| --- | --- | --- | --- | --- | --- | --- | --- | --- |
| Band  No. | Lane  % | Fragment length  (base pair) | Band  No. | Lane  % | Fragment length  (base pair) | Band  No. | Lane  % | Fragment length  (base pair) |
| 1 | 7.97 | 4000.000 | 1 | 19.73 | 1726.362 | 1 | 16.25 | 1006.568 |
| 2 | 7.23 | 3000.000 | 2 | 54.71 | 1466.109 | 2 | 9.30 | 972.801 |
| 3 | 7.71 | 2000.000 | 3 | 25.56 | 1259.550 | 3 | 10.78 | 939.783 |
| 4 | 3.28 | 1500.000 |  |  |  | 4 | 6.94 | 894.429 |
| 5 | 2.35 | 1200.000 |  |  |  | 5 | 13.45 | 833.009 |
| 6 | 23.02 | 1000.000 |  |  |  | 6 | 8.92 | 807.964 |
| 7 | 1.14 | 900.000 |  |  |  | 7 | 1.05 | 740.659 |
| 8 | 8.24 | 800.000 |  |  |  | 8 | 7.97 | 557.666 |
| 9 | 4.66 | 700.000 |  |  |  | 9 | 16.61 | 408.394 |
| 10 | 15.12 | 600.000 |  |  |  | 10 | 8.72 | 336.480 |
| 11 | 2.91 | 500.000 |  |  |  |  |  |  |
| 12 | 2.39 | 400.000 |  |  |  |  |  |  |
| 13 | 0.65 | 300.000 |  |  |  |  |  |  |
| 14 | 0.38 | 200.000 |  |  |  |  |  |  |
| 15 | 12.38 | 100.000 |  |  |  |  |  |  |
| 16 | 0.57 | 50.000 |  |  |  |  |  |  |

**Table S9.** Variation in DNA bands of SNPs-treated (a) and untreated (b) cells of *E. coli*

| Ref. Band | (a) | | | (b) | | |
| --- | --- | --- | --- | --- | --- | --- |
|  | Band No. | Lane % | Fragment length  (base pair) | Band No. | Lane % | Fragment length  (base pair) |
| Band 1 | 1 | 19.73 | 1726.362 |  |  |  |
| Band 2 | 2 | 54.71 | 1466.109 |  |  |  |
| Band 3 | 3 | 25.56 | 1259.550 |  |  |  |
| Band 4 |  |  |  | 1 | 16.25 | 1006.568 |
| Band 5 |  |  |  | 2 | 9.30 | 972.801 |
| Band 6 |  |  |  | 3 | 10.78 | 939.783 |
| Band 7 |  |  |  | 4 | 6.94 | 894.429 |
| Band 8 |  |  |  | 5 | 13.45 | 833.009 |
| Band 9 |  |  |  | 6 | 8.92 | 807.964 |
| Band 10 |  |  |  | 7 | 1.05 | 740.659 |
| Band 11 |  |  |  | 8 | 7.97 | 557.666 |
| Band 12 |  |  |  | 9 | 16.61 | 408.394 |
| Band 13 |  |  |  | 10 | 8.72 | 336.480 |

**Table S10.** Matrix data of DNA pattern parameters of SNPs-treated (a) and untreated (b) cells of *E. coli*

| Ref. Band | Fragment length  (base pair) | (a) | (b) |
| --- | --- | --- | --- |
| Band 1 | 1726.362 | 1 | 0 |
| Band 2 | 1466.109 | 1 | 0 |
| Band 3 | 1259.550 | 1 | 0 |
| Band 4 | 1006.568 | 0 | 1 |
| Band 5 | 972.801 | 0 | 1 |
| Band 6 | 939.783 | 0 | 1 |
| Band 7 | 894.429 | 0 | 1 |
| Band 8 | 833.009 | 0 | 1 |
| Band 9 | 807.964 | 0 | 1 |
| Band 10 | 740.659 | 0 | 1 |
| Band 11 | 557.666 | 0 | 1 |
| Band 12 | 408.394 | 0 | 1 |
| Band 13 | 336.480 | 0 | 1 |
